# *EPM2 AIP1* immunohistochemistry as a surrogate of promoter methylation analysis in endometrial carcinoma

**DOI:** 10.1007/s00428-025-04132-3

**Published:** 2025-06-05

**Authors:** Sonia Gatius, Marta Vaquero, Oliver Scheiber, Ana Velasco, Dolors Cuevas, Karl Kashofer, Maria Santacana, Núria Eritja, Sigurd Lax, Xavier Matias-Guiu

**Affiliations:** 1 Hospital Universitari Arnau de Vilanova. IRBLleida, Universitat de Lleida, Av Rovira Roure 80, CIBERONC, Lleida, 25198 Spain; 2 Department of Pathology, General Hospital Graz II, Styrian Hospital Corporation, Graz, Austria; 3 Diagnostic & Research Institute of Pathology, Medical University of Graz, Graz, Austria; 4 Johannes Kepler University Linz, Linz, Austria; 5 Scientific and Technical Service of Immunohistochemistry, Lleida Institute for Biomedical Research Dr. Pifarré Foundation, IRBLleida, Hospital Universitari Arnau de Vilanova, Av. Alcalde Rovira Roure, 80, Lleida, 25198 Spain; 6 Oncologic Pathology Group, Department of Medicine and Surgery, Biomedical Research Institute of Lleida (IRBLleida), University of Lleida, Av. Rovira Roure 80, Lleida, 25198 Spain

**Keywords:** Endometrial cancer, MLH-1 methylation, Lynch syndrome, EPM2AIP1

## Abstract

Mismatch repair (MMR) status in endometrial carcinoma (EC) is crucial for diagnosis, prognosis, treatment, and Lynch syndrome pre-screening. *MLH1* loss is the most frequent cause of MMR deficiency and usually by promoter hypermethylation. We tried to confirm the role of *EPM2 AIP1* immunohistochemistry as a surrogate of *MLH1* promoter methylation in EC. Case series from two different institutions were analyzed by comparable methods using immunohistochemistry for MMR proteins and *EPM2 AIP1,* and pyrosequencing for *MLH1* methylation. In the first series of 70 cases, concordance was 100%, after reassessing three cases with methylation scores close to cut-off, by tumor cell enrichment. In the second series of 29 *MLH1*-deficient ECs, concordance was 96.5%, while in the control group of 30 MMR-proficient EC, one *MLH1*-positive case was *EPM2 AIP1*-negative. *EPM2 AIP1* immunoreactivity was qualitatively superior in curettages and biopsies compared to hysterectomy. We conclude that *EPM2 AIP1* immunohistochemistry is a good surrogate for *MLH1* promoter methylation analysis, cost-effective with short turnaround time, but needs attention regarding preanalytical handling, normal tissue contamination, or low tumor percentage.

## Introduction

Around 30% of endometrial carcinomas (EC) show mismatch repair deficiency (MMRd) and consecutive microsatellite instability (MSI) [1–7]. Testing for mismatch repair (MMR) status in EC patients is important since MMRd is a prognostic marker within the molecular classification of EC, predictive for response to immune checkpoint inhibitors, a diagnostic marker for endometrioid type EC, and useful to identify Lynch syndrome patients [8–10]. Lynch syndrome is an autosomal dominant cancer predisposition disorder resulting from constitutional pathogenic sequence variants affecting one of the DNA mismatch repair genes *MLH1*, *MSH2*, *MSH6*, or *PMS2,* and frequently associated with EC and carcinomas of the colon and other organs. The inherited pathogenic sequence variant affects one allele, and for tumor development, a somatic pathogenic variant or a deletion of the second allele (“second hit”) is required [10]. In sporadic EC, somatic biallelic silencing by promoter hypermethylation of *MLH1* is the most common mechanism leading to MMRd/MSI. In most guidelines, *MLH1* methylation analysis is requested for all ECs showing loss of *MLH1* immunoreactivity. The presence of *MLH1* promoter hypermethylation virtually rules out the possibility of Lynch syndrome, with a few reported exceptions. Several *MLH1* methylation tests are commercially available, such as methylation-specific PCR, MLPA, or pyrosequencing [11–15]. These techniques are quite complex and time-consuming, and, therefore, often not available in pathology departments. Therefore, a surrogate marker for methylation analysis, most likely by immunohistochemistry, is highly wanted to facilitate the diagnostic workup process of *MLH1*-negative, MMRd EC.

Recently, *EPM2 AIP1* immunohistochemistry (IHC) was suggested as a potential surrogate of promoter methylation in EC [16]. *MLH1* acts as a bi-directional promoter, with the *EPM2 AIP1* gene positioned on the antisense strand. Methylation of the *MLH1* promoter region leads to the transcriptional silencing of both *MLH1* and *EPM2 AIP1* genes. However, additional validation studies are required to support the incorporation of this test in clinical practice.

The aim of this study is to confirm the role of *EPM2 AIP1 IHC* as a potential surrogate of *MLH1* promoter methylation in EC, which we tested in independent series from 2 different institutions.

## Materials and methods

### Samples selection

The first series (from Lleida) consisted of 70 EC cases with *MLH1*-negative ECs by IHC (56 low-grade endometrioid carcinomas and 14 high-grade endometrioid carcinomas; 55 from pipelle biopsy, 15 from hysterectomy specimens) and 30 *MLH1*-positive EC (23 low-grade endometrioid carcinomas, 4 high-grade endometrioid carcinomas, and 3 serous carcinomas, all of them from pipelle biopsy) interpreted as controls. All samples were from patients diagnosed and treated at the Hospital Universitari Arnau de Vilanova de Lleida, Spain (HUAV). Tumor samples were available at IRBLLEIDA Biobank, registered in the National Registry of Biobanks of Instituto de Salud Carlos III (B.00682). All samples were collected following the current regulations in the biomedical research law, the Royal Decree of Biobanks RD 1716/2011, the European Regulation 2016/679, and the organic law LOPD-GDD 3/2018 on the protection of personal data, and all patients signed an informed consent. The study was approved by local Ethics Committees, in accordance with the Declaration of Helsinki.

The second series (from Graz) consisted of 30 *MLH1*-negative EC (24 low-grade endometrioid carcinomas, 5 high-grade endometrioid carcinomas, 1 dedifferentiated carcinoma; 24 from curettage or pipelle, 6 from hysterectomy specimens), and 30 *MLH1*-positive EC (28 low-grade endometrioid carcinomas, 1 high-grade endometrioid carcinoma, 1 clear cell carcinoma; 25 from curettage or pipelle, 5 from hysterectomy specimens) from the Department of Pathology, Hospital Graz II, Styrian Hospital Corporation, an academic teaching hospital of the Medical University of Graz, Austria. The cases were retrieved from the files after a search in the laboratory information system.

### Mismatch repair protein immunohistochemistry

The MMR/MSI status was assessed for all cases by immunohistochemistry using either a 4-antibody or a 2-antibody approach.

In the first series, whole-section FFPE was cut into 3 microns and dried at 65 °C. The deparaffinization, rehydration, and antigen recovery pretreatments were then performed in the PT-LINK pretreatment module (DAKO) at 95 °C for 20 min with 50 × Tris/EDTA buffer at pH 9. Endogenous peroxidase was inhibited before staining the sections. The following antibodies were used: *MLH1* (clone ES05, Agilent DAKO), *MSH2* (clone FE11, Agilent DAKO), *MSH6* (clone EP49, Agilent DAKO), and *PMS2* (clone EP51, Agilent DAKO). After incubation, the reaction was visualized using the EnVision Detection Kit (DAKO), using diaminobenzidine as the substrate. The sections were counterstained with hematoxylin. In the second series, mismatch repair protein IHC was performed using the 2-antibody approach on 2-µm paraffin sections. In a first step, all cases were analyzed for *MSH6* (clone SP93, Roche/Ventana) and *PMS2* (clone A16-4, Roche/Ventana) immunoreactivity, and cases with loss of *PMS2* immunoreactivity were further analyzed for *MLH1* (clone M1, Roche/Ventana). The procedure was performed on a Ventana Benchmark Ultra™ with a pretreatment for 64 (*MSH6*), 92 (*PMS2*), and 32 (*MLH1*) min at 100 (*MSH6*, *PMS2*) and 95 °C (*MLH1*), respectively, using buffer CC1. The primary antibody was incubated for 12 min at 36 °C (*MSH6*), 24 min at room temperature, and 32 min at 36 °C (*MLH1*), respectively. For detection, OptiView DAB was used with (*MLH1*, *PMS2*) or without (*MSH6*) amplification. An on-slide positive control was used on all slides.

Loss of expression was considered in cases showing nuclear negativity in tumor cells with a positive internal control in the stromal tissue. An on-slide positive tonsillar tissue control was used on all slides.

### EPM2 AIP1 immunohistochemistry

In both series, the *EPM2 AIP1* mouse monoclonal antibody (Clone: OTI2 A2, OriGene Technologies) was used on paraffin sections (cut at 3 and 2 µm, respectively). Nuclear *EPM2 AIP1* immunoreactivity in benign cells of endometrium and myometrium (glandular epithelial, stromal, smooth muscle, and inflammatory cells) served in addition as an internal positive control. Cases without nuclear immunoreactivity for *EPM2 AIP1* were considered negative and hypermethylated, respectively, whereas cases with any positive nuclear staining were considered positive and unmethylated, respectively. The percentage of nuclear staining was documented.

The first series used a PT-LINK pretreatment module (DAKO) at 95 °C for 20 min with 50 × Tris/EDTA buffer at pH 9 for deparaffinization, rehydration, and antigen recovery pretreatment. The primary antibody was diluted at 1:100 and incubated for 30 min at room temperature. Endogenous peroxidase was inhibited before staining. After incubation, the reaction was visualized using the EnVision Detection Kit (DAKO), using diaminobenzidine as the substrate. Sections were counterstained with hematoxylin and assessed by two pathologists (S.G. and X.M.).

The second series used a Ventana Benchmark Ultra platform with a pretreatment at 100 °C for 20 min with CC2 buffer. The primary antibody was diluted at 1:250 and incubated for 32 min at 36 °C. Endogenous peroxidase was blocked before the procedure. For detection, OptiView DAB was used without amplification. Sections were counterstained with hematoxylin and assessed by two pathologists (O.S. and S.F.L.).

### Promoter methylation analysis

In both series, *MLH1* methylation analysis was performed by a pyrosequencing (PSQ) assay (Qiagen) on all cases with *MLH1* + *PMS2*-deficient immunohistochemistry, both laboratories being accredited according to EN ISO 15189 for this procedure.

In the first series, bisulfite-treated DNA (Methyl Code Bisulfite Conversion Kit, Thermo Fisher) was amplified and pyrosequenced to address the methylation level of 5 CpG sites within the region comprised between − 209 and − 181 from the transcription start site of the *MLH1* gene.

For the second series, *MLH1* methylation analysis was performed at the Molecular Pathology Laboratory of the Diagnostic & Research Institute of Pathology of the Medical University of Graz, which is accredited according to EN ISO 15189. DNA was extracted from 5-μm-thick FFPE tissue sections and treated with sodium bisulfite for the conversion of unmethylated cytosine. Subsequently, the promoter regions between positions − 248 and − 178 from the transcription start site (TSS) and containing 8 CpG sites were amplified by PCR using a specific primer set developed by PyroMark Assay Design software (Qiagen GmbH, Germany). The generated Amplicon was sequenced using Pyromark Q24 (Qiagen GmbH, Germany), and the methylation status of the *MLH1* promoter was determined by analysis of the sequence with PyroMark CpG software (Qiagen GmbH, Germany).

For both series, a sample was classified as methylated when the mean of all five cytosines was higher than 10% of methylation, and cases between 5 and 10% of methylation were reassessed.

### Next generation sequencing

Tumor sequencing by a panel that included *MLH1* was performed in two EC *MLH1*-deficient, unmethylated tumors. Genomic DNA was isolated from samples using the EZ2 DNA Extraction Kit (Qiagen), following the manufacturer’s protocol. DNA libraries were prepared using the Agilent XT H2 Library Prep Kit, with fragmentation, adapter ligation, and PCR amplification steps optimized for downstream Sequencing. Libraries were sequenced on the MGI G99 platform (2 × 100 bp paired-end reads) to achieve an average coverage of > 100 ×. For bioinformatic analysis, raw sequencing data were processed and analyzed using SeqOne for variant calling, annotation, and quality control.

## Results

### Methylation analysis and correlation with EPM2 AIP1 immunohistochemistry (Fig. 1; Table 1)

**Fig. 1:**
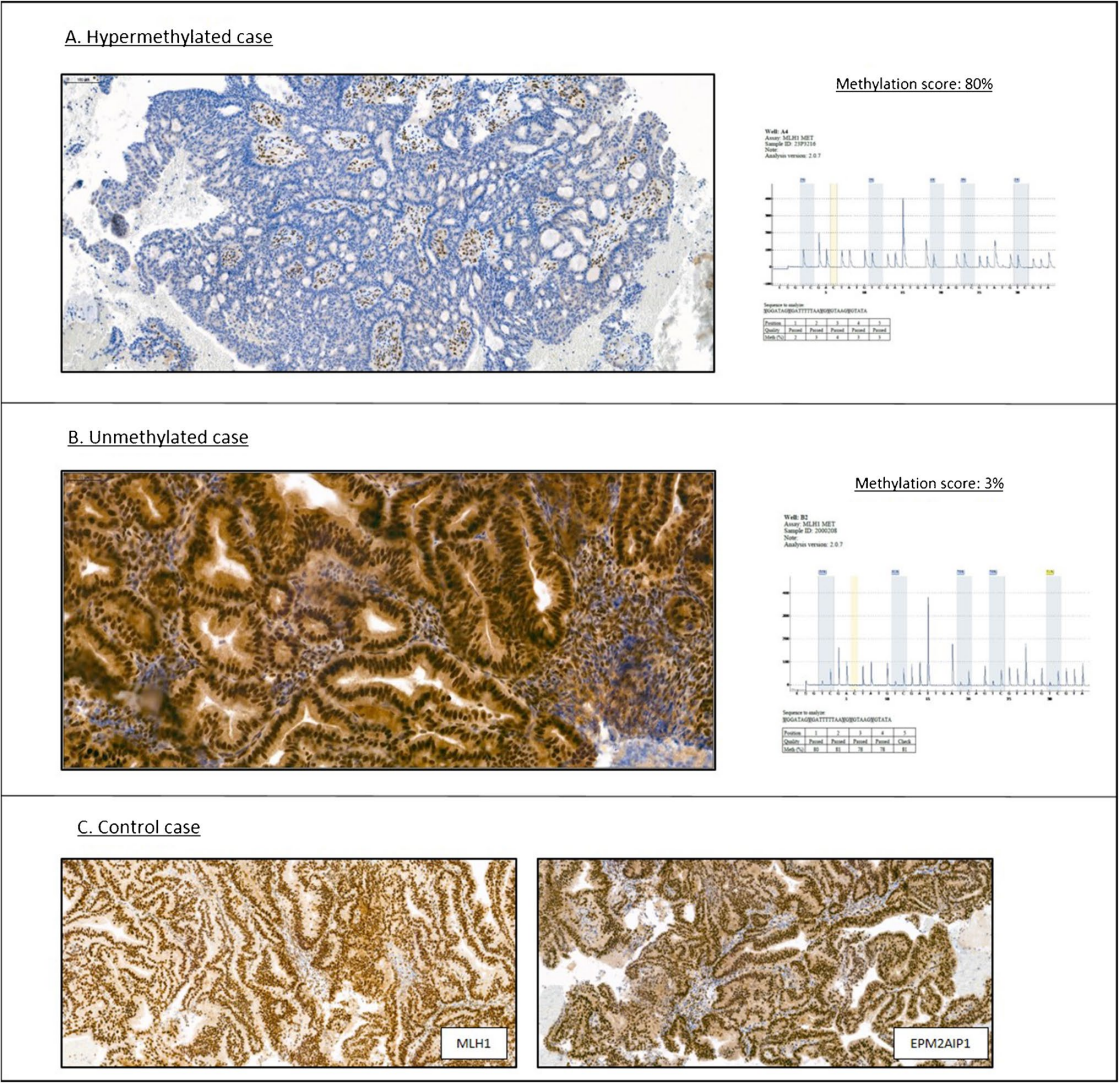
Representative images of *EPM2 AIP1* immunohistochemistry and promoter methylation chart of one example of EC with *MLH1* hypermethylation **A**, and one example of EC without *MLH1* hypermethylation **B**. Representative images of *MLH1* and *EPM2 AIP1* immunohistochemistry in one EC with normal expression of both proteins (control case) **C**

**Table 1:** Clinicopathological features (histologic type and FIGO2023 staging) as well as results of *MLH1* expression, methylated status of *MLH1* by pyrosequencing, and *EPM2 AIP1* immunohistochemical expression of cases of the first series (HUAV), and the second series (GRAZ2) of cases

Hospital	Case	Histological type	FIGO 2023	*MLH1* expression	Methylation status	Methylation score	*EPM2 AIP1* expression
LLEIDA	1	EEC2	NA	Negative	Hypermethylated	48.20%	Negative
LLEIDA	2	EEC2	IIB	Negative	Unmethylated	3%	Positive
LLEIDA	3	EEC2	IB	Negative	Hypermethylated	77.20%	Negative
LLEIDA	4	EEC1	IB	Negative	Hypermethylated	60.80%	Negative
LLEIDA	5	EEC1	IB	Negative	Hypermethylated	64.80%	Negative
LLEIDA	6	EEC3	IC	Negative	Hypermethylated	31%	Negative
LLEIDA	7	EEC1	IIIC2	Negative	Hypermethylated	76%	Negative
LLEIDA	8	EEC1	IA2	Negative	Hypermethylated	68.60%	Negative
LLEIDA	9	EEC1	IA2	Negative	Hypermethylated	62%	Negative
LLEIDA	10	EEC1	IIIC2	Negative	Hypermethylated	40.40%	Negative
LLEIDA	11	EEC1	IIIC2	Negative	Hypermethylated	27.80%	Negative
LLEIDA	12	EEC1	NA	Negative	Hypermethylated	49.80%	Negative
LLEIDA	13	EEC2	NA	Negative	Hypermethylated	72.60%	Negative
LLEIDA	14	EEC1	IIIA	Negative	Hypermethylated	57.40%	Negative
LLEIDA	15	EEC1	NA	Negative	Hypermethylated	77.80%	Negative
LLEIDA	16	EEC1 + HCA	IA1	Negative	Hypermethylated	60.00%	Negative
LLEIDA	17	EEC1	IA1	Negative	Hypermethylated	18.80%	Negative
LLEIDA	18	EEC1	IIA	Negative	Hypermethylated	38.20%	Negative
LLEIDA	19	EEC1	IIIC1	Negative	Hypermethylated	79.60%	Negative
LLEIDA	20	EEC1	IA2	Negative	Hypermethylated	51.80%	Negative
LLEIDA	21	EEC3	NA	Negative	Hypermethylated	37.60%	Negative
LLEIDA	22	EEC2	IB	Negative	Hypermethylated	83%	Negative
LLEIDA	23	EEC1	IA2	Negative	Hypermethylated	65.20%	Negative
LLEIDA	24	EEC1	IIB	Negative	Hypermethylated	43.20%	Negative
LLEIDA	25	EEC2	IA1	Negative	Hypermethylated	81.00%	Negative
LLEIDA	26	EEC1	IIIC2	Negative	Hypermethylated	41.20%	Negative
LLEIDA	27	EEC3	IA2	Negative	Hypermethylated	84.40%	Negative
LLEIDA	28	EEC2	IIIB	Negative	Hypermethylated	75.40%	Negative
LLEIDA	29	EEC1	IIIA	Negative	Hypermethylated	84.80%	Negative
LLEIDA	30	EEC1	IVC	Negative	Hypermethylated	65%	Negative
LLEIDA	31	EEC2	IA2	Negative	Hypermethylated	82.80%	Negative
LLEIDA	32	EEC + HCA	IA1	Negative	Unmethylated	9%	Positive
LLEIDA	33	EEC3	IIC	Negative	Hypermethylated	66.80%	Negative
LLEIDA	34	EEC1 + HCA	IA1	Negative	Hypermethylated	41.60%	Negative
LLEIDA	35	EEC1	IA1	Negative	Hypermethylated	21.80%	Negative
LLEIDA	36	EEC3	IIC	Negative	Hypermethylated	83.80%	Negative
LLEIDA	37	EEC1	NA	Negative	Hypermethylated	19.60%	Negative
LLEIDA	38	EEC2	IIIC2	Negative	Hypermethylated	77.80%	Negative
LLEIDA	39	EEC1	IA2	Negative	Hypermethylated	68.80%	Negative
LLEIDA	40	EEC2	IIIC2	Negative	Hypermethylated	47.60%	Negative
LLEIDA	41	EEC2	NA	Negative	Hypermethylated	63.40%	Negative
LLEIDA	42	EEC3	NA	Negative	Hypermethylated	64.80%	Negative
LLEIDA	43	EEC1	IA1	Negative	Hypermethylated	84.20%	Negative
LLEIDA	44	EEC1	IA2	Negative	Hypermethylated	27.20%	Negative
LLEIDA	45	EEC1	IA2	Negative	Hypermethylated	77%	Negative
LLEIDA	46	EEC1	IIIC1	Negative	Hypermethylated	51.20%	Negative
LLEIDA	47	EEC3	NA	Negative	Hypermethylated	89.60%	Negative
LLEIDA	48	EEC1	IIIC1	Negative	Hypermethylated	76.80%	Negative
LLEIDA	49	EEC3	IIC	Negative	Hypermethylated	71.60%	Negative
LLEIDA	50	EEC1	IA2	Negative	Hypermethylated	68.60%	Negative
LLEIDA	51	EEC1	IA2	Negative	Hypermethylated	72.40%	Negative
LLEIDA	52	EEC2	NA	Negative	Hypermethylated	52%	Negative
LLEIDA	53	EEC3	IIC	Negative	Unmethylated	2%	Positive
LLEIDA	54	EEC2	IA2	Negative	Unmethylated	2.40%	Positive
LLEIDA	55	EEC3	IIC	Negative	Unmethylated	3%	Positive
LLEIDA	56	EEC2	IA2	Negative	Hypermethylated	51.40%	Negative
LLEIDA	57	EEC3	IIC	Negative	Hypermethylated	81.60%	Negative
LLEIDA	58	EEC3	IIC	Negative	Hypermethylated	73.20%	Negative
LLEIDA	59	EEC2	IA2	Negative	Hypermethylated	69.80%	Negative
LLEIDA	60	EEC3	IIIB1	Negative	Hypermethylated	62.40%	Negative
LLEIDA	61	EEC1	IA2	Negative	Hypermethylated	19.60%	Negative
LLEIDA	62	EEC2	IA2	Negative	Hypermethylated	32%	Negative
LLEIDA	63	EEC2	IB	Negative	Hypermethylated	66.80%	Negative
LLEIDA	64	EEC2	IIIA1	Negative	Hypermethylated	71.40%	Negative
LLEIDA	65	EEC3	NA	Negative	Hypermethylated	91.40%	Negative
LLEIDA	66	EEC1	IA2	Negative	Hypermethylated	68.60%	Negative
LLEIDA	67	EEC1	IIIC1	Negative	Hypermethylated	59.60%	Negative
LLEIDA	68	EEC1	IIA	Negative	Hypermethylated	67.60%	Negative
LLEIDA	69	EEC1	NA	Negative	Hypermethylated	33.20%	Negative
LLEIDA	70	EEC1	NA	Negative	Hypermethylated	33.20%	Negative
LLEIDA	71	EEC1	IA2	Positive	NA	NA	Positive
LLEIDA	72	EEC2	IA2	Positive	NA	NA	Positive
LLEIDA	73	EEC	IA2	Positive	NA	NA	Positive
LLEIDA	74	EEC1	IA2	Positive	NA	NA	Positive
LLEIDA	75	EEC1	IA1	Positive	NA	NA	Positive
LLEIDA	76	EEC1	IB	Positive	NA	NA	Positive
LLEIDA	77	EEC1	IIIA	Positive	Unmethylated	2.60%	Positive
LLEIDA	78	EEC1	IA2	Positive	NA	NA	Positive
LLEIDA	79	EEC3	IIC	Positive	NA	NA	Positive
LLEIDA	80	EEC1	IIA	Positive	NA	NA	Positive
LLEIDA	81	SC	IIC	Positive	NA	NA	Positive
LLEIDA	82	EEC2	IA2	Positive	NA	NA	Positive
LLEIDA	83	SC	IIC	Positive	NA	NA	Positive
LLEIDA	84	EEC3	IIC	Positive	NA	NA	Positive
LLEIDA	85	EEC1	IA2	Positive	NA	NA	Positive
LLEIDA	86	EEC	IA1	Positive	NA	NA	Positive
LLEIDA	87	EEC3	IIC	Positive	NA	NA	Positive
LLEIDA	88	EEC1	IIIB2	Positive	Unmethylated	2.40%	Positive
LLEIDA	89	EEC3	IIC	Positive	NA	NA	Positive
LLEIDA	90	SC	IIC	Positive	NA	NA	Positive
LLEIDA	91	EEC3	IIC	Positive	NA	NA	Positive
LLEIDA	92	EEC1	IB	Positive	NA	NA	Positive
LLEIDA	93	EEC1	IA2	Positive	NA	NA	Positive
LLEIDA	94	EEC1	IB	Positive	NA	NA	Positive
LLEIDA	95	EEC1	IA2	Positive	NA	NA	Positive
LLEIDA	96	EEC3	IIC	Positive	NA	NA	Positive
LLEIDA	97	EEC1	IIB	Positive	NA	NA	Positive
LLEIDA	98	EEC1	IA2	Positive	NA	NA	Positive
LLEIDA	99	EEC1	IA2	Positive	NA	NA	Positive
LLEIDA	100	EEC1	IA2	Positive	NA	NA	Positive
GRAZ2	101	EEC2	NA	Negative	Hypermethylated	15.60%	Positive
GRAZ2	102	EEC3	NA	Negative	Hypermethylated	36.00%	Negative
GRAZ2	103	EEC2	NA	Negative	Hypermethylated	35.00%	Negative
GRAZ2	104	EEC2	NA	Negative	Hypermethylated	17.40%	Negative
GRAZ2	105	EEC1	NA	Negative	Hypermethylated	15.00%	Negative
GRAZ2	106	EEC2	NA	Negative	Hypermethylated	68.50%	Negative
GRAZ2	107	EEC2	NA	Negative	Hypermethylated	41.30%	Negative
GRAZ2	108	EEC1	NA	Negative	Hypermethylated	48.90%	Negative
GRAZ2	109	EEC2	NA	Negative	Hypermethylated	73.50%	Negative
GRAZ2	110	EEC1	NA	Negative	Hypermethylated	19.30%	Negative
GRAZ2	111	EEC2	NA	Negative	Hypermethylated	27.00%	Negative
GRAZ2	112	EEC1	NA	Negative	Hypermethylated	30.50%	Negative
GRAZ2	113	EEC1	NA	Negative	Hypermethylated	82.00%	Negative
GRAZ2	114	EEC3	NA	Negative	Hypermethylated	66.10%	Negative
GRAZ2	115	EEC1	NA	Negative	Hypermethylated	61.90%	Negative
GRAZ2	116	EEC1	NA	Negative	Hypermethylated	58.10%	Negative
GRAZ2	117	EEC1	NA	Negative	Hypermethylated	67.10%	Negative
GRAZ2	118	EEC1	NA	Negative	Hypermethylated	52.70%	Negative
GRAZ2	119	EEC1	NA	Negative	Hypermethylated	18.40%	Negative
GRAZ2	120	EEC1	NA	Negative	Hypermethylated	21.20%	Negative
GRAZ2	121	EEC1	NA	Negative	Hypermethylated	89.90%	Negative
GRAZ2	122	EEC3	NA	Negative	Hypermethylated	7.40%	Negative
GRAZ2	123	EEC1	NA	Negative	Hypermethylated	70.00%	Negative
GRAZ2	124	EEC3	NA	Negative	Hypermethylated	58.50%	Negative
GRAZ2	125	EEC1	NA	Negative	Hypermethylated	55.40%	Negative
GRAZ2	126	EEC1	NA	Negative	Hypermethylated	50.80%	Negative
GRAZ2	127	EEC + SC + CC	NA	Negative	Hypermethylated	71.50%	Negative
GRAZ2	128	EEC3	NA	Negative	Hypermethylated	85.00%	Negative
GRAZ2	129	EEC1	NA	Negative	Hypermethylated	65.80%	Negative
GRAZ2	130	EEC2	NA	Positive	NA	NA	Positive
GRAZ2	131	EEC2	NA	Positive	NA	NA	Positive
GRAZ2	132	EEC1	NA	Positive	NA	NA	Positive
GRAZ2	133	EEC1	NA	Positive	NA	NA	Positive
GRAZ2	134	EEC1	NA	Positive	NA	NA	Positive
GRAZ2	135	EEC3	NA	Positive	NA	NA	Positive
GRAZ2	136	EEC1	NA	Positive	NA	NA	Positive
GRAZ2	137	EEC1	NA	Positive	NA	NA	Positive
GRAZ2	138	EEC1	NA	Positive	NA	NA	Positive
GRAZ2	139	EEC1	NA	Positive	NA	NA	Positive
GRAZ2	140	EEC2	NA	Positive	NA	NA	Positive
GRAZ2	141	EEC1	NA	Positive	NA	NA	Positive
GRAZ2	142	EEC1	NA	Positive	NA	NA	Positive
GRAZ2	143	EEC2	NA	Positive	NA	NA	Positive
GRAZ2	144	EEC2	NA	Positive	NA	NA	Positive
GRAZ2	145	EEC1	NA	Positive	NA	NA	Positive
GRAZ2	146	EEC1	NA	Positive	NA	NA	Positive
GRAZ2	147	EEC2	NA	Positive	NA	NA	Negative
GRAZ2	148	EEC1	NA	Positive	NA	NA	Positive
GRAZ2	149	EEC1	NA	Positive	NA	NA	Positive
GRAZ2	150	EEC1	NA	Positive	NA	NA	Positive
GRAZ2	151	EEC2	NA	Positive	NA	NA	Positive
GRAZ2	152	EEC2	NA	Positive	NA	NA	Positive
GRAZ2	153	EEC1	NA	Positive	NA	NA	Positive
GRAZ2	154	CC	NA	Positive	NA	NA	Positive
GRAZ2	155	EEC1	NA	Positive	NA	NA	Positive
GRAZ2	156	EEC1	NA	Positive	NA	NA	Positive
GRAZ2	157	EEC1	NA	Positive	NA	NA	Positive
GRAZ2	158	EEC1	NA	Positive	NA	NA	Positive
GRAZ2	159	EEC1	NA	Positive	NA	NA	Positive
GRAZ2	160	EEC1	NA	Negative	NA/shortage of tumor cells	NA	Negative

**Fig. 2:**
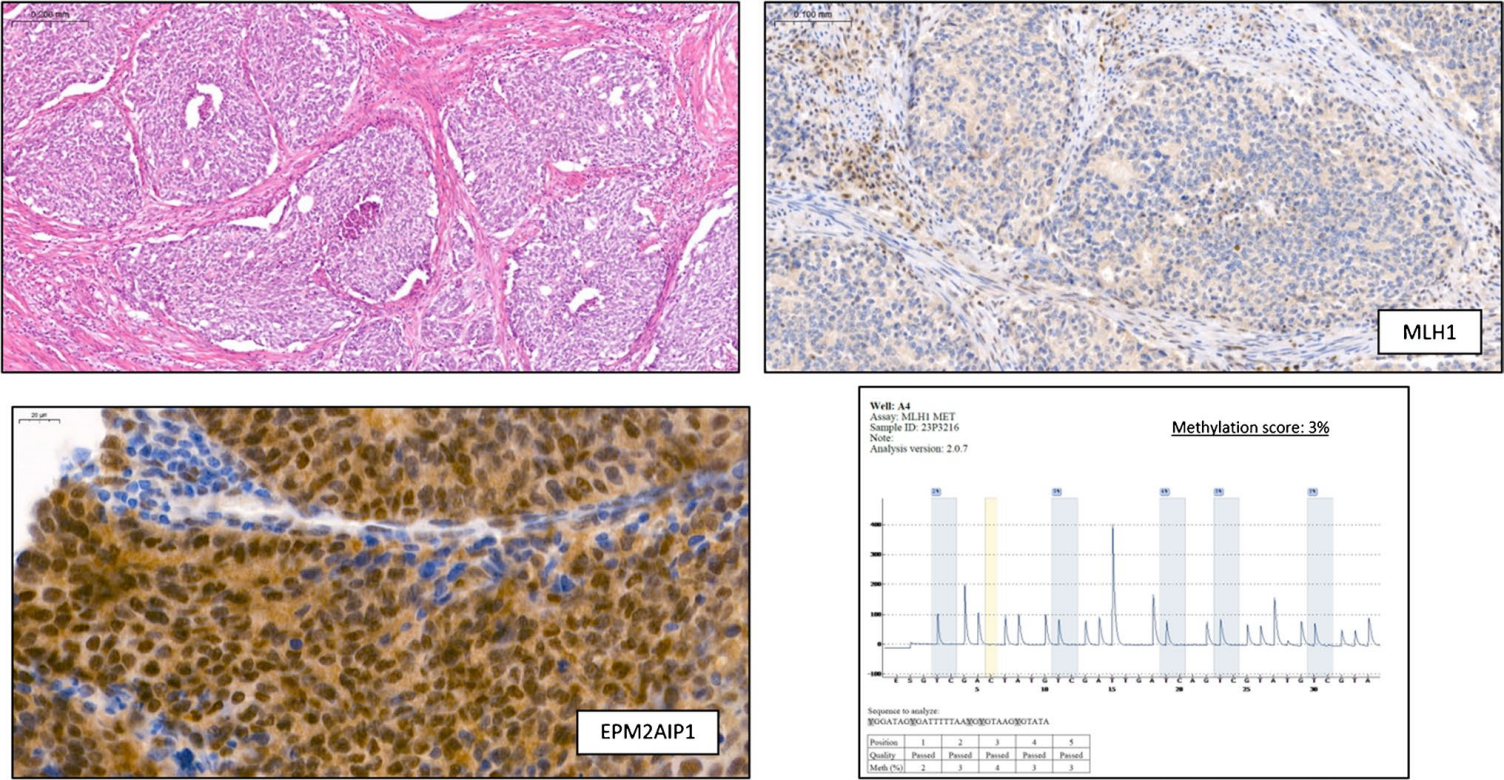
Representative images of an EC with *MLH1* deficiency, absence of *MLH1* promoter methylation, and positive *EPM2 AIP1* immunostaining. The tumor exhibited biallelic somatic alteration of *MLH1*; one pathogenic variant in *MLH1* (NM_000249.4): p.Glu633 Ter (c.1897G > T), classified as a stop-gained mutation, truncating the *MLH1* protein, as well as loss of heterozygosity (LOH) detected in chromosomal region 3p22.2-q27.1

**Fig. 3:**
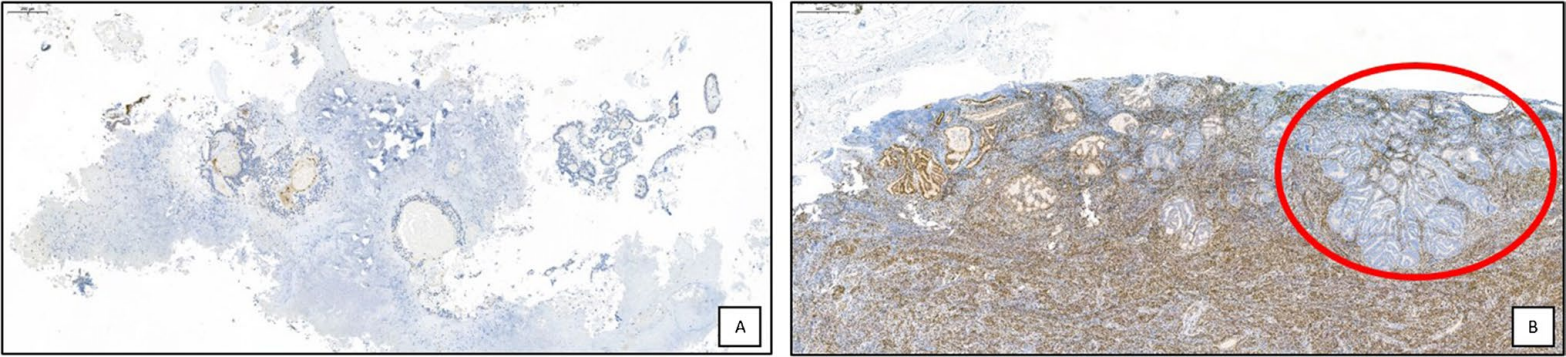
Representative images of an EC with *MLH1* with a methylation score close to the cut-off. The initial biopsy **A** showed negative *EPM2 AIP1* immunostaining and a methylation score of 9.8. In the surgical specimen **B**, *EPM2 AIP1* immunostaining was negative, but the methylation score increased to 60% after tumor microdissection

In the first series, pyrosequencing-based methylation analysis of the *MLH1* promoter identified 64 hypermethylated cases out of 70 total *MLH1*-deficient ECs (range 13.8 to 89.6%). All hypermethylated cases exhibited negative nuclear expression of *EPM2 AIP1*. The methylation test also showed six unmethylated cases out of 70, and five of them showed *EPM2 AIP1* nuclear expression, with one discrepant case with a borderline scoring (9.8%) and negative *EPM2 AIP1* nuclear expression. Tumor sequencing data on the *MLH1* gene was available in two of the 5 MLH1-deficient, unmethylated tumors, and both of them exhibited a somatic *MLH1* mutation (Fig. 2). Three cases were further analyzed because their methylation scores were close to the cut-off (Fig. 3). Two of these were endometrioid carcinomas arising from atypical endometrial hyperplasia. Their methylation scores were 13.8 and 14.4%, percentages close to the 10% threshold for hypermethylation. In both cases, *EPM2 AIP1 IHC* was negative in carcinoma areas but positive in hyperplastic regions. In the third case (the discordant one), the methylation analysis was performed on a curettage biopsy, which had a low tumor percentage and extensive necrosis. The methylation score was 9.8%, yet *EPM2 AIP1* showed negative nuclear expression. IHC analysis of *EPM2 AIP1* was repeated on the surgical specimen in all discrepant cases, yielding the same negative result. Subsequently, macrodissection of the tumor areas was conducted in each of these three cases, and pyrosequencing was repeated. After tumor enrichment by macrodissection, the methylation score increased in all cases (scorings of 60%, 81%, and 41.6%). Consequently, the discrepant case was reclassified as hypermethylated. Following the re-evaluation of the discrepant case, the concordance between the two techniques was 100%. In summary, results show concordance between *MLH1* promoter methylation analysis by pyrosequencing and *EPM2 AIP1* IHC, with 100% *EPM2 AIP1* negativity in the 70 cases that exhibited *MLH1* promoter methylation by pyrosequencing.

In the second series, pyrosequencing-based methylation analysis of the *MLH1* promoter revealed a mean of all five cytosines higher than 10% of methylation in 28 *MLH1* immunonegative cases (range 7.4–89.9%, mean 48.6%) with one case between 5 and 10% of methylation (7.4%). In one case with complete loss of *EPM2 AIP1,* methylation analysis was not possible due to too small amount of tumor tissue left in the paraffin block and no further tumor tissue available. One *MLH1* + *PMS2* immunonegative case with a methylation score of 15.6% and microsatellite instability by PCR was positive for *EPM2 AIP1*. For this case, methylation analysis and immunohistochemistry for *MLH1* were repeated on the hysterectomy specimen and revealed a methylation score of 23% and *MLH1* immunonegativity. Overall agreement between the methylation assay and *EPM2 AIP1* immunohistochemistry was, therefore, 93.1% with a false negative rate of 6.9% at a cut-off of 10%, and 96.5% with a false negative rate of 3.5% at a cut-off of 5%, respectively.

### EPM2 AIP1 immunohistochemistry in MLH1 positive EC

In the first series, all 30 cases of the *MLH1*-positive control group exhibited positive nuclear expression of *EPM2 AIP1* (100% concordance between *MLH1* and *EPM2 AIP1* immunoreactivity), with no differences between pipelle material and hysterectomy specimens.

In the second series, one out of 30 MMR-proficient ECs with intact *MLH1* and *PMS2* immunoreactivity showed complete loss of *EPM2 AIP1* immunoreactivity. The agreement between *MLH1* and *EPM2 AIP1* immunoreactivity was, therefore, 96.7% with a false negative rate of 3.3%. *EPM2 AIP1* immunoreactivity was different between curettage/pipelle material and hysterectomy specimens with respect to staining intensity and background staining. A diffuse strong immunoreactivity in more than 80–90% of the tumor cells, like in the non-neoplastic stromal cells, was only found in curettage/pipelle tissue, whereas tumor tissue from hysterectomy specimens showed weaker staining in the tumor cell nuclei compared to the non-neoplastic stromal cells, heterogeneous staining intensity, and in 50% of the cases, cytoplasmic staining (table).

## Discussion

In this validation study, we were able to show in two independent series of EC a high concordance between *MLH1* promoter methylation and *EPM2 AIP1* immunohistochemistry, as well as between *MLH1* and *EPM2 AIP1* immunohistochemistry. The concordance between the analyzed techniques was 100% in one series, in the other greater than 95%. This is along with a previous retrospective study on 119 EC, which suggested *EPM2 AIP1* immunohistochemistry as a viable surrogate for *MLH1* methylation in EC [16]. In their study, Mrkonjic et al. found a concordance between *EPM2 AIP1* IHC and *MLH1* promoter methylation in 95% of cases, with 94.5% sensitivity and a positive predictive value of 98.1% [16]. The *EPM2 AIP1* and *MLH1* genes share the same promoter region, and methylation of this promoter region leads to transcriptional silencing of both genes, and both have also been found to be silenced by promoter methylation in colon and gastric cancers [17–19].

*MLH1* promoter methylation is an epigenetic phenomenon leading to transcriptional silencing of the gene. In sporadic tumors, biallelic silencing by *MLH1* promoter hypermethylation is the most common mechanism driving MSI/MMRd. *MLH1* promoter methylation can be analyzed through various molecular methods, such as pyrosequencing or methylation-specific PCR, among others [11–14]. While these techniques are extensively validated, particularly in colon cancer, they are complex, costly, and time-consuming, and they are not universally available in pathology departments. These techniques have also some differences. Pyrosequencing has the advantage of providing not only a qualitative assessment (methylated or unmethylated), but also a quantitative value of methylation status. The vast majority of *MLH1* unmethylated ECs have very low scoring values, while the values for *MLH1* methylated tumors are very high. This is an opportunity for paying attention to the ECs cases that have scores close to the cut-off point. Since there is no universally validated scoring for endometrial carcinoma, we used here a 10%. For diagnostic analysis, the lab at the Medical University Graz uses a 5% cut-off. It is important to double-check whether the tumor cell content in the selected paraffin block is appropriate, or whether there is subclonality, which is an important issue in EC. Subclonal MSI/MMRd is found in 10% of MSI/MMRd ECs, in which the majority of tumor cells are MMR proficient [20–22]. There is some evidence that tumors with subclonal MSI/MMRd are associated with worse prognosis compared to other MSI/MMRd ECs without subclonality [23]. Analysis of *MLH1* promoter methylation in these cases may be challenging if precise microdissection and appropriate tumor content assessment is not carefully performed.

Identifying *MLH1* methylated EC may, therefore, also be interesting at the prognostic level. There is evidence that *MLH1* methylated ECs are associated with adverse prognostic factors in comparison with MMRd/MSI EC caused by mutations in the MMR genes [24–30]. In addition, there are differences at the mutational level, and with respect to the composition of the tumor microenvironment.

In our first series, the results showed high concordance between promoter methylation and loss of *EPM2 AIP1* expression, with 100% agreement, since all *MLH1* methylated cases exhibited loss of *EPM2 AIP1* expression. Reevaluation of the tumors with scorings close to the cut-off of 10% revealed two endometrioid carcinomas arising from atypical endometrial hyperplasia with *EPM2 AIP1* positivity in hyperplastic areas and loss of expression in carcinoma areas. The third case showed a low tumor percentage in the biopsy and extensive necrotic areas, and a methylation score of 9.8%. In these three cases, tumor areas with *EPM2 AIP1* negativity resulted as *MLH1* methylated after tumor enrichment through microdissection. In cases with low tumor cell percentage, IHC appears to outperform molecular testing as it allows evaluation in a specific histological context. In the control group of 30 EC with preserved *MLH1* expression, *EPM2 AIP1* was positive throughout.

In the second series of cases, one *MLH1* methylated EC showed diffuse positive immunoreactivity for *EPM2 AIP1,* leading to a concordance between *MLH1* promoter methylation analysis and *EPM2 AIP1* immunohistochemistry of 96.5%. The results are accordingly with the previous study, which reported a similar case. The control group of the second series contained another discrepant case with loss of *EPM2 AIP1* immunoreactivity despite intact MLH1 immunoreactivity. These outliers in the *EPM2 AIP1* assessment are in accordance with the previous study and show that the agreement is very good but not 100%. It is currently unclear which molecular mechanism leads to expression of *EPM2 AIP1* in *MLH1* methylated EC, as well as lack of *EPM2 AIP1* expression potentially by downregulation in the absence of *MLH1* methylation. Nevertheless, *EPM2 AIP1* seems to work well as a putative surrogate for *MLH1* promoter methylation in most cases, and it is important to be aware of potential pitfalls.

Aside from the rare events of *EPM2 AIP1* positivity in *MLH1* methylated EC and *EPM2 AIP1* negativity in *MLH1* non-methylated EC, the second series unraveled additional diagnostic hints, particularly the weaker staining for *EPM2 AIP1* in hysterectomy specimens due to suboptimal fixation. Whereas tissue harvested by curettage or pipelle is usually immediately fixed, hysterectomy specimens are often neither immediately submitted to pathology labs nor opened and properly fixed. Therefore, endometrial tumors often undergo delayed fixation, leading to an accelerated degradation, particularly of non-structural proteins. This seems to be the case with *EPM2 AIP1*, too.

Interestingly, a recent article by Challa et al. showed that *EPM2 AIP1* immunohistochemistry was not as good in colorectal cancer as in EC [31]. The authors found a loss of *EPM2 AIP1* immunoreactivity only in 64% of cases with *MLH1* promoter methylation. On the other side, 33% of cases without *MLH1* promoter methylation were negative for *EPM2 AIP1*. Of note, two *MLH1*-germline mutated tumors without *MLH1* promoter methylation showed loss of *EPM2 AIP1* immunoreactivity. In their study, *EPM2 AIP1* loss was 64% sensitive and 67% specific for *MLH1* promoter hypermethylation, with an accuracy of 64%. It is worth mentioning, however, that MSI/MMRd colorectal carcinomas and MSI/MMRd EC show molecular differences with respect to the level of instability and the co-existence between MSI/MMRd and *BRAF* V600E mutations in colorectal carcinomas [32–36].

We conclude that *EPM2 AIP1* immunohistochemistry serves as a reliable, although not 100% concordant, surrogate for *MLH1* promoter methylation analysis. Our findings suggest that *EPM2 AIP1* immunohistochemistry offers greater specificity, as it allows the evaluation of histological slides, thereby minimizing the risk of false negative methylation tests caused by normal tissue contamination or a low tumor cell percentage. This is particularly important in a setting where microdissection is problematic. The quality of *EPM2 AIP1* immunohistochemistry seems to be particularly dependent on preanalytical conditions such as prompt fixation, and therefore assessment on curettage and pipelle material seems to be superior to hysterectomy specimens. Additionally, immunohistochemistry is cost-effective, widely accessible in most pathology laboratories, and has a fast turnaround time compared to a PCR- or sequencing-based technique. Molecular methylation assays may be preserved for uncertain and equivocal cases as a second method.

## Data Availability

Data available within the article or its supplementary materials.
